# Iridium-based probe for luminescent nitric oxide monitoring in live cells

**DOI:** 10.1038/s41598-018-30991-9

**Published:** 2018-08-20

**Authors:** Chun Wu, Ke-Jia Wu, Tian-Shu Kang, Hui-Min David Wang, Chung-Hang Leung, Jin-Biao Liu, Dik-Lung Ma

**Affiliations:** 1 Department of Chemistry, Hong Kong Baptist University, Kowloon Tong, Hong Kong, China; 2 State Key Laboratory of Quality Research in Chinese Medicine, Institute of Chinese Medical Sciences, University of Macau, Macao, China; 3 Graduate Institute of Biomedical Engineering, National Chung Hsing University, Taichung Taiwan, China; 4 School of Metallurgical and Chemical Engineering, Jiangxi University of Science and Technology, Ganzhou, China

## Abstract

Nitric oxide (NO) is an intra- and extracellular messenger with important functions during human physiology process. A long-lived luminescent iridium(III) complex probe **1** has been designed and synthesized for the monitoring of NO controllably released from sodium nitroprusside (SNP). Probe **1** displayed a 15-fold switch-on luminescence in the presence of SNP at 580 nm. The probe exhibited a linear response towards SNP between 5 to 25 μM with detection limit at 0.18 μM. Importantly, the luminescent switch-on detection of NO in HeLa cells was demonstrated. Overall, complex **1** has the potential to be applied for NO tracing in complicated cellular environment.

## Introduction

Nitric oxide (NO) plays an irreplaceable role in multiple processes of various physiology and pathology pathways, such as regulating vasodilatation, relaxation and immunization response, as well as the cardiovascular, peripheral and central nervous systems^1–7^. Uncontrollable NO secretion is highly linked with the production of reactive nitrogen species (RNS) that can cause health issues including inflammation, endothelial dysfunction, neurodegeneration diseases or even cancer^8,9^. However, the function mechanisms and complicated physiological involvement of NO are still not fully understood. The availability for rapid and selective NO monitoring turns out to be one of the key requirements for further investigation towards NO.

An attractive approach to detect NO in real time is with fluorescent probes^10^. A number of NO fluorescent organic probes have been developed for bioimaging^11–13^, including a number of *o*-phenylenediamine-based probes by the group of Nagano (Table S1)^14–16^. A frequently-used moiety for trapping NO is the electron-rich *o*-diaminophenyl functionality, as it is an effective quencher of fluorescence via photoinduced electron transfer (PET). Mechanistically, the reaction of NO with the *o*-diaminophenyl group under aerobic conditions forms the corresponding benzotriazole moiety, leading to the abrogation of PET quenching and the restoration of fluorsecence^17^. However, although several NO probes based on organic dyes have been proposed^18^, the development of transition metal-based NO probes with advantageous characteristics, such as large Stokes shift, good photostability, long luminescence lifetime, and high intracellular retention^19–23^, remains a challenge.

The Lippard group has developed several Cu(II)-based NO probes^24–26^. The mechanism of these Cu(II)-based probes relies on the generation of a diamagnetic Cu(I) species triggered by NO reduction, which abolishes the fluorescence quenching accompanied with the paramagnetic Cu(II) center. Yuan’s group has also reported an Eu(III) bearing luminescence probe that is selective towards NO^27^. Additionally, an Ir(III) probe has been developed for endogenous NO imaging, which displayed good selectivity for NO tracking in the mitochondria of living cells^28^.

**Figure 1:**
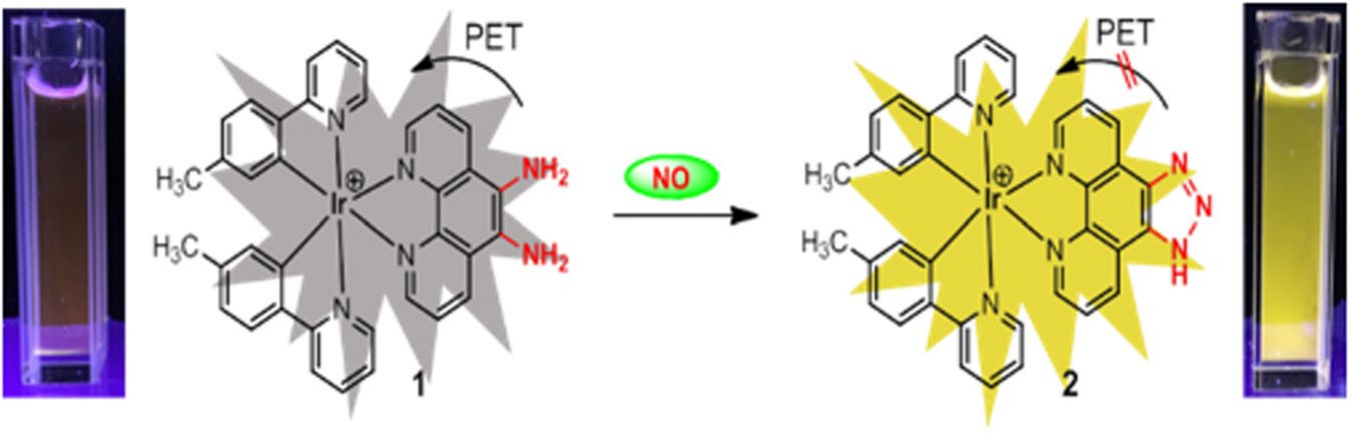
Mechanism of NO detection by complex **1**.

Based on our current interest in the development of luminescent iridium(III) complex probes^29–34^, we attempted to develop an iridium(III) complex platform for NO determination using SNP as the source of NO. We designed and synthesized an iridium(III) complex **1**, incorporating the *o*-diamine group in the phenanthroline N^N ligand, and with two 2-(*p*-tolyl)pyridine (tpy) units as C^N co-ligands. We envisioned that **1** could act as a NO probe through the reaction of the *o*-diamino groups with NO to form the triazole **2**. This reaction would inhibit the PET quenching effect from the *o*-diamino groups, leading to an increased switch-on response in the presence of NO (Fig. 1).

## Results

### Photophysical properties of complex 1

Complex **1** is readily generated from the organometallated dimer ([Ir(tpy)_2_Cl]_2_) and 1,10-phenanthroline-5,6-diamine (Fig. S1). High resolution mass spectrometry (HRMS) and ^1^H, ^13^C NMR spectroscopy were employed for the characterization of complex **1** (Fig. S2). With complex **1** in hand, we next investigated the photophysical characteristics of complex **1**. Complex **1** exhibited a 265 ns lifetime (Table S2), which is typical of phosphorescence iridium(III) complexes, while organic dyes usually possess lifetimes within nanosecond regime. Importantly, the long lifetime of such iridium(III) scaffolds could allow them to be distinguished in autofluorescent samples via time-resolved luminescence spectroscopy (TRES). Moreover, **1** showed a maximal emission wavelength at 608 nm after excited at 355 nm, giving a calculated Stokes shift for about 253 nm (Table S2), which is much greater than the Stokes shifts generally shown by organic dyes.

### Signal response of **1** to NO

**Figure 2:**
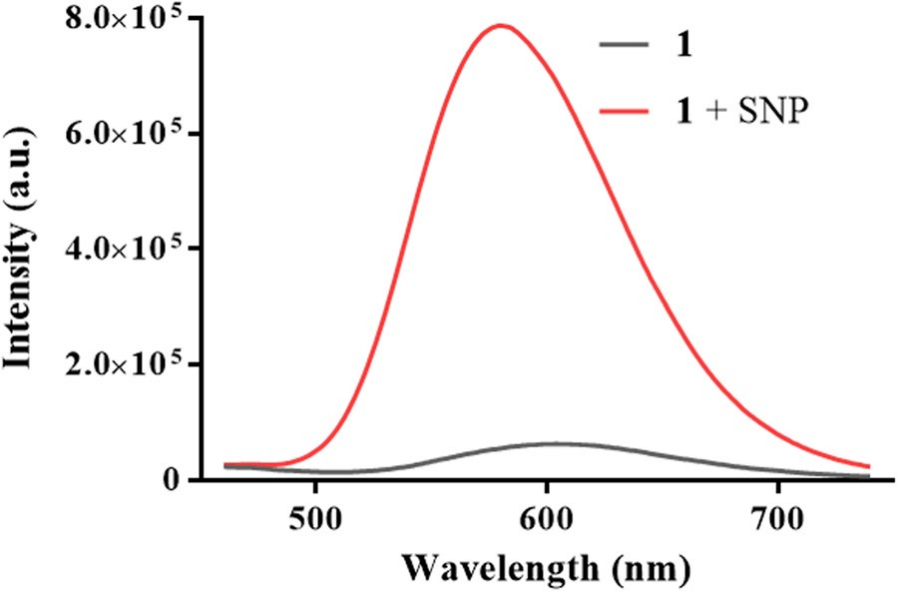
Emission spectra of complex **1** (5 μM) in the absence and presence of SNP (25 μM) in DMSO/PBS (9:1, v/v).

As endogenous NO production is usually uncontrollable and ambiguous in certain cases, an exogenous NO source such as sodium nitroprusside (SNP)^35,36^ is commonly used to understand the role of NO in pathologic and physiologic pathways. We initially investigated the emission response of **1** towards NO generated from SNP after UV light irradiation for one minute. In the absence of SNP, **1** displayed very weak luminescence in a 9:1 blend of dimethyl sulfoxide (DMSO) and PBS buffer (50 mM, pH = 7.4). However, upon the addition of SNP, the luminescence signal of **1** was enhanced significantly by around 15-fold (Fig. 2), which was largely attributed to the accelerated release of NO from SNP under UV light^37^.

**Figure 3:**
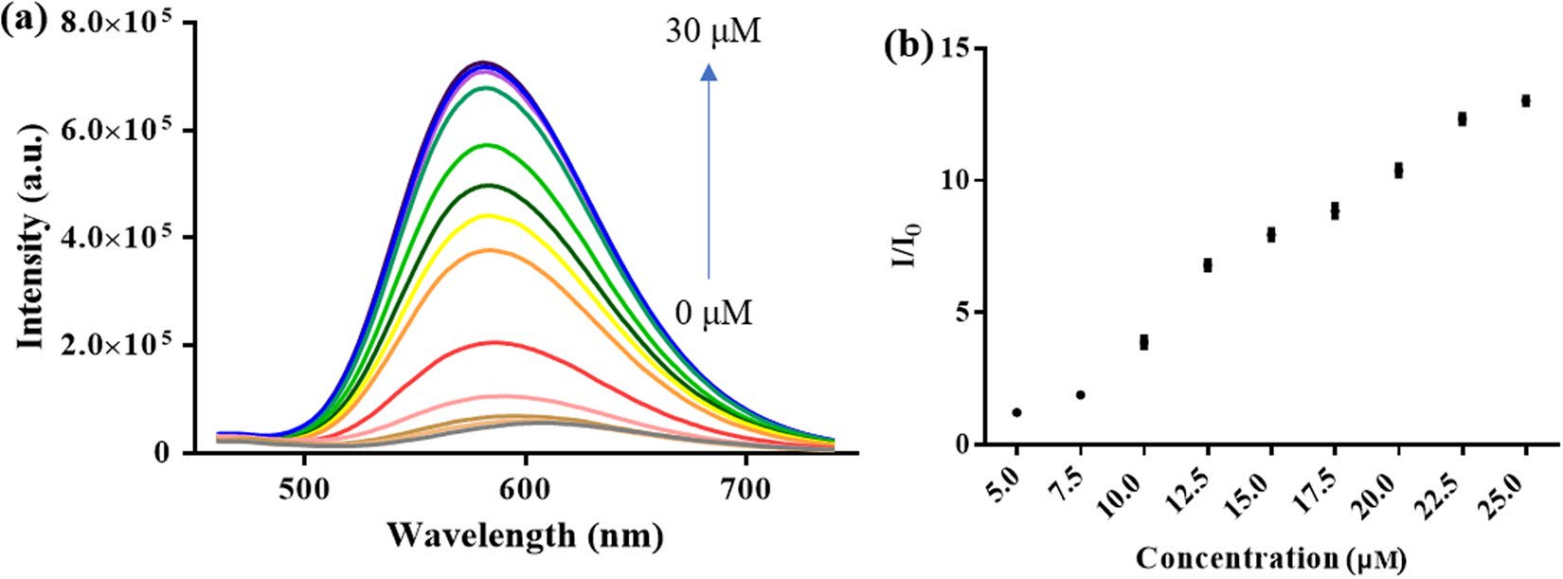
(**a**) Luminescence spectra of complex **1** (5 μM) in DMSO/PBS buffer (9:1, v/v, 50 mM, pH = 7.4) with increasing concentration of SNP: 0, 2.5, 5.0, 7.5, 10.0, 12.5, 15.0, 17.5, 20.0, 22.5, 25.0, 27.5, 30.0 μM). (**b**) Luminescence enhancement ratio of complex **1** upon increasing SNP concentration.

We next investigated the luminescent behavior of **1** to SNP in solvent systems containing various proportions of DMSO and PBS buffer. Our probe displayed the most significant luminescence enhancement in DMSO/buffer (9:1, v/v), whereas the luminescence response of the probe was decreased as the percentage of DMSO in solution was reduced (Fig. S3). We also examined other organic solvents such as DMSO, dimethyl formamide (DMF), acetonitrile (ACN), acetone, tetrahydrofuran (THF), ethanol (EtOH) and methanol (MeOH) for the detection system (with 10% PBS buffer solution) and discovered that DMSO offered the most optimal probe response (Fig. S4). In an emission titration experiment, complex **1** (5 μM) displayed an increasing luminescence intensity with increasing concentration of SNP (Fig. 3a). A linear correlation (R^2^ = 0.980) was observed from 5 to 25 µM of SNP, and a detection limit of 0.18 μM was measured (Fig. 3b) based on the 3σ method. To validate the reaction-based mechanism of the assay, HRMS analysis was performed on the reaction mixture. The results indicated the production of triazole **2** at m/z = 750.1918 (expected m/z: 750.1952), suggesting that the reaction between complex **1** with NO took place (Fig. S5). Complex **2** exhibited a maximum emission wavelength at 584 nm, which represented a blue shift of 24 nm compared to complex **1** (608 nm). Importantly, complex **2** showed much stronger luminescence intensity compared to complex **1**. Moreover, complex **2** showed a stronger UV-Vis absorbance profile and a longer luminescence lifetime compared to complex **1** (Fig. S6).

### Selectivity of complex **1** for NO

**Figure 4:**
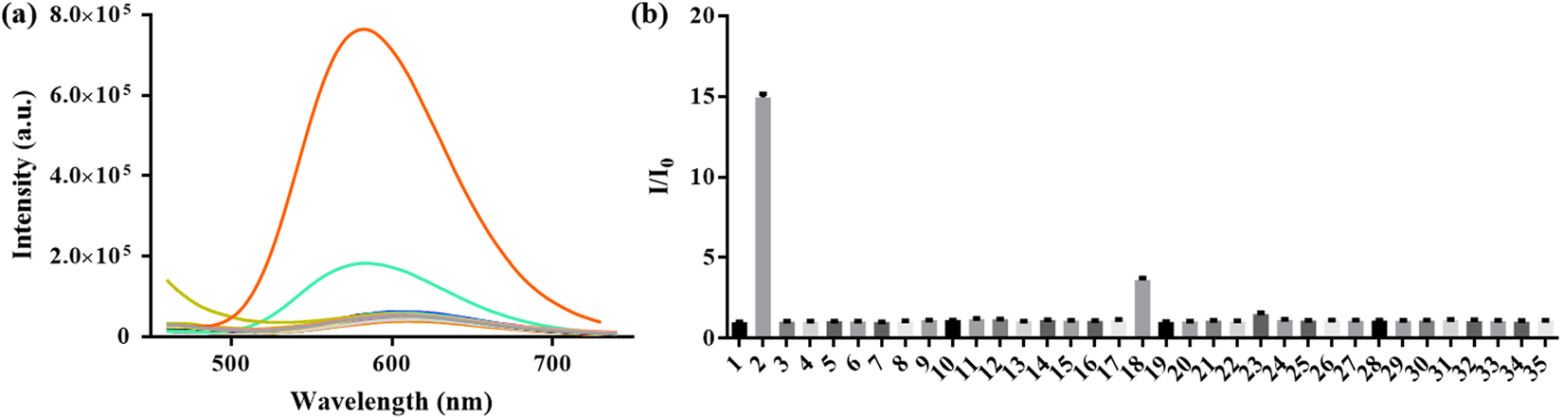
(**a**) Luminescence intensity change at 580 nm in the presence of various targets: (1) complex **1** (5 μM), (2) SNP, (3) Met, (4) Pro, (5) Trp, (6) Val, (7) Ala, (8) Asp, (9) Tyr, (10) Arg, (11) Glu, (12) Cys, (13) GSH, (14) Mg^2+^, (15) Na^+^, (16) Ca^2+^, (17) NO_3_^−^, (18) NO_2_^−^, (19) H_2_O_2_, (20) HClO, (21) Lys, (22) Gly, (23) His, (24) Phe, (25) Leu, (26) Asn, (27) Ile, (28) Ser, (29) Thr, (30) Gln, (31) Cl^−^, (32) CO_3_^2−^, (33) HCO_3_^−^, (34) HPO_4_^2−^, (35) H_2_PO_4_^−^. λ_ex_ = 355 nm. (**b**) The bars show the relative luminescence intensity change of complex **1** and further addition of various interferences at 580 nm.

The selectivity of **1** (5 μM) for SNP was tested over other possible biologically relevant species (25 μM), including reactive oxygen species (H_2_O_2_ and HClO), nitrogen species (NO_2_^−^ and NO_3_^−^), amino acids (Cys, GSH, Glu, Arg, Tyr, Asp, Tyr, Val, Trp, Pro, Ala, Met, Lys, Gly, His, Phe, Leu, Asn, Ile, Ser, Thr and Gln), metal ions (Na^+^, Mg^2+^ and Ca^2+^) and anions (Cl^−^, CO_3_^2−^, HCO_3_^−^, HPO_4_^2−^, H_2_PO_4_^−^). Negligible luminescence response of **1** toward these species were observed, with the exception of NO_2_^−^ which produced a 3-fold intensity enhancement (Fig. 4). In contrast, only SNP displayed a very strong turn-on luminescence at 580 nm, indicating the high selectivity of **1** towards SNP. This selectivity could be attributed to the unique reaction between the *o*-diamine functionality of **1** with NO, producing the triazole unit. The high selectivity of complex **1** for NO renders it as a reliable probe for tracing NO in physiological environments.

### Application of SNP detection assay in live cells

Encouraged by the performance of complex **1** for the *in vitro* detection of SNP, the cytotoxicity of complex **1** was measured in HeLa cells and normal liver LO2 cells (Fig. S7). The results revealed that complex **1** exhibited negligible toxicity towards either HeLa cells or LO2 cells at 100 μM. This indicates that complex **1** is relatively nontoxic to cells, making it suitable for cell imaging applications.

**Figure 5:**
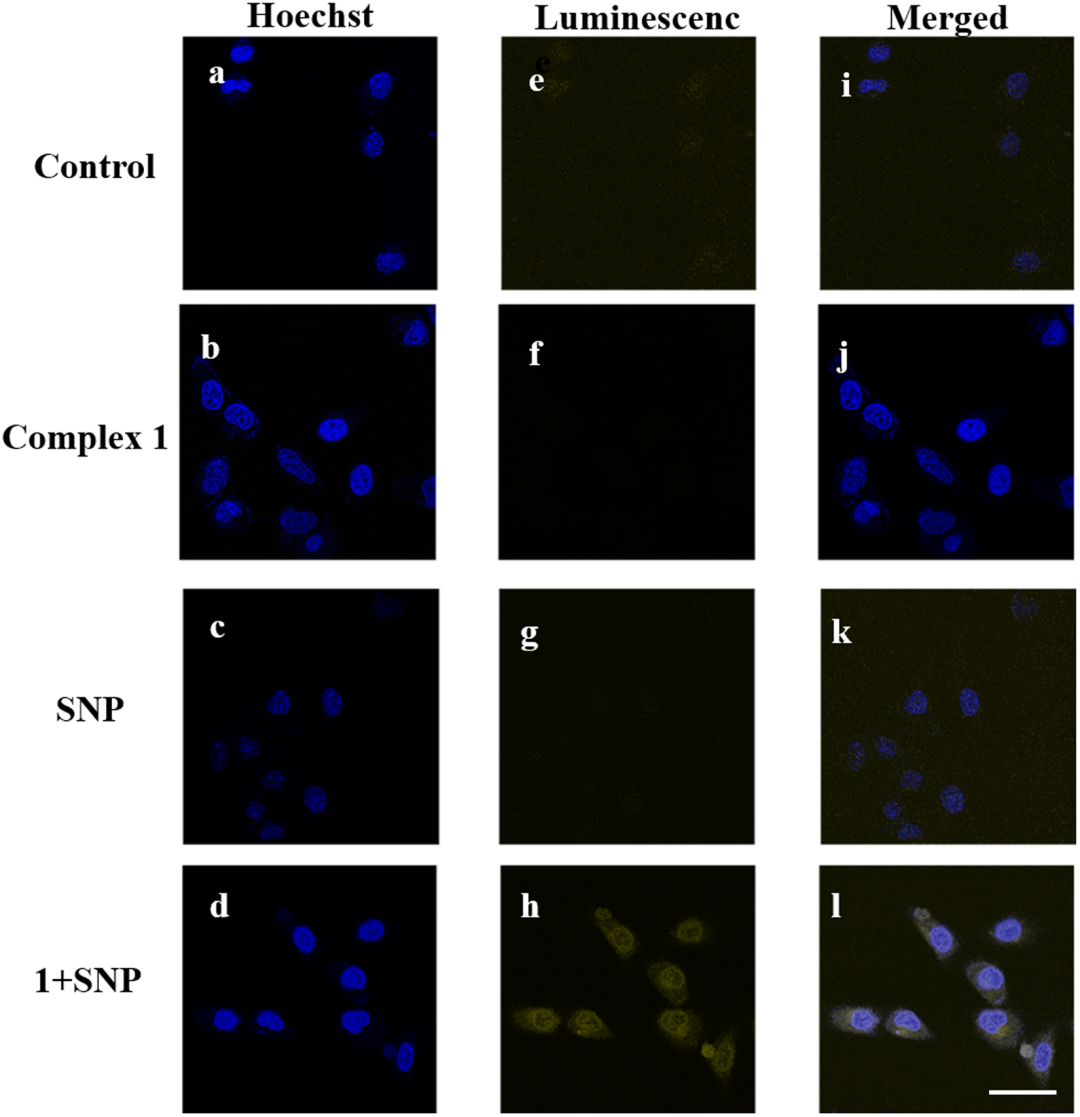
Confocal imaging of HeLa cells incubated with complex **1** (10 μM) with SNP (100 μM) for 3 h at 37 °C and UV irradiation for 10 min. Excitation was at 405 nm, and luminescence images were recorded from 570 to 640 nm. Nuclei were stained with 5 μg/mL Hoechst for 5 min. The scale bar is 50 μm.

We next explored whether complex **1** could be employed for the tracing of NO in HeLa cells. Upon the addition of complex **1** (10 μM) only or SNP (100 μM) only, no significant luminescence could be observed (Fig. 5) even after UV light irradiation for 10 min. However, a strong yellow luminescence could be observed in the presence of both complex **1** and SNP under the same condition. This suggests that complex **1** could be used to image NO in living cells, with the further potential to demonstrate the involvement of NO in cellular reactions. In order to examine the accumulation of complex **1** in cells, an inductively-coupled plasma mass spectrometry (ICP-MS) assay was performed (Fig. S8). As shown in Fig. S8, HeLa cells incubated with complex **1** showed a significant enhancement of iridium content in the cellular environment, suggesting the successful transportation of complex **1** into HeLa cells.

Considering the feasibility for intracellular sensing of NO using complex **1**, the intracellular response of complex **1** upon addition of different concentrations of SNP (0–100 μM) was further investigated. As shown in Fig. S9, HeLa cells displayed a luminescence that was mainly generated in cytoplasm at lower concentrations of SNP (10–50 μM). However, upon increasing of SNP concentration to 100 μM, a detectable luminescence generated from complex **1** could also be observed in the nuclear area, suggesting that higher concentrations of SNP might facilitate detectable NO monitoring in both the cytoplasm and nucleus of HeLa cells.

Moreover, the intracellular luminescence intensities of HeLa cells treated with complex **1** (10 μM) and SNP (100 μM) for different times (1, 3 and 6 h) were monitored using fluorescent microscopy (Fig. S10). The luminescence of the cells increased over time and was predominantly localized in the cytoplasm at 1 h, before spreading to the whole cell at 3 and 6 h, indicating the feasibility for NO detection within 6 h in the cellular environment.

## Discussion

In this paper, we have successfully designed and synthesized an iridium(III) probe **1** and used it as a turn-on chemosensor for NO monitoring. **1** bears an *o*-diamine group in the N^N donor ligand, which allows it to act as a recognition unit for NO. In the presence of SNP, **1** experienced about 15-fold emission increase at 580 nm. Compared with typical organic dyes, **1** displayed a long lifetime luminescence and a wide Stokes shift. We expect that probe **1** could offer a versatile scaffold for assisting the mechanism investigations of NO in biological processes.

## Methods

### Nitric oxide detection

Sodium nitroprusside (SNP) was dissolved in water to achieve a 1 M stock concentration. Afterwards, different concentrations of SNP were added to DMSO/PBS buffer (9:1, v/v, pH = 7.4) containing complex **1** (5 μM) in a cuvette for 1 min irradiation under UV light at 365 nm. Luminescence emission spectra were recorded on a PTI QM-1 spectrofluorometer (Photo Technology International, Birmingham, NJ) at 25 °C, with the slits for both excitation and emission set at 2.5 nm. UV-Vis absorption spectra were recorded on a Cary UV-300 spectrophotometer (double beam).

### Confocal imaging

Cells were seeded into a glass-bottomed dish (35 mm dish with 20 mm well). After 12 h, cells were incubated with complex **1** and SNP for the indicated time periods or concentrations, followed by UV light irradiation for 10 min and further washing with phosphate-buffered saline three times. The luminescence imaging of complexes in cells was carried out by a Leica TCS SP8 confocal laser scanning microscope system. The excitation wavelength was 405 nm.

## Electronic supplementary material

Electronic supplementary information
