# Supplementary material for: Iridium-based probe for luminescent nitric oxide monitoring in live cells
Source: Sci Rep. 2018 Aug 20;8:12467. doi: 10.1038/s41598-018-30991-9 (PMC6102254; doi:10.1038/s41598-018-30991-9)
Supplement: Supplementary file 1 — Electronic supplementary information [file 41598_2018_30991_MOESM1_ESM.docx]

Electronic Supporting Information

**Iridium-based probe for luminescent nitric oxide monitoring in live cells**

Chun Wu^1^, Ke-Jia Wu^2^, Tian-Shu Kang^2^, Hui-Min David Wang^3^, Chung-Hang Leung^2^, Jin-Biao Liu^1,4^* and Dik-Lung Ma^1^*

^1^ Department of Chemistry, Hong Kong Baptist University, Kowloon Tong, Hong Kong.

*^2^ State Key Laboratory of Quality Research in Chinese Medicine, Institute of Chinese Medical Sciences, University of Macau, Macao.*

*^3^* *Graduate Institute of Biomedical Engineering, National Chung Hsing University, Taichung, 402, Taiwan.*

*^4^ School of Metallurgical and Chemical Engineering, Jiangxi University of Science and Technology, Ganzhou, China.*

** Corresponding authors.*

*E-mail addresses: edmondma@hkbu.edu.hk (Dr. Dik‐Lung Ma) and liujbgood@hotmail.com (Dr. Jin-Biao Liu).*

**TABLE OF CONTENTS**

1. **Materials and general experiments** **S2**
2. **Experimental details**  **S2**
3. **Supporting information** **S3-S6**
4. **References**  **S6**
5. **Materials and general experiments**

**Materials.** Reagents, unless specified, were purchased from Sigma Aldrich (St. Louis, MO) and used as received. Iridium chloride hydrate (IrCl_3_·xH_2_O) was purchased from Precious Metals Online (Australia). Human cervical cancer cell line, HeLa, was purchased from ATCC.

**General experiments**. Mass spectrometry was performed at the Mass Spectroscopy Unit at the Department of Chemistry, Hong Kong Baptist University, Hong Kong (China). Deuterated solvents for NMR purposes were obtained from Armar and used as received.

^1^H and ^13^C NMR were recorded on a Bruker Avance 400 spectrometer operating at 400 MHz (^1^H) and 100 MHz (^13^C). ^1^H and ^13^C chemical shifts were referenced internally to solvent shift (dimethyl sulfoxide (DMSO)-*d*_6_: ^1^H δ 2.50, ^13^C δ 39.5). Chemical shifts (δ) are quoted in ppm, the downfield direction being defined as positive. Uncertainties in chemical shifts are typically ±0.01 ppm for ^1^H and ±0.05 for ^13^C. Coupling constants are typically ± 0.1 Hz for ^1^H-^1^H and ±0.5 Hz for ^1^H-^13^C couplings. The following abbreviations are used for convenience in reporting the multiplicity of NMR resonances: s, singlet; d, doublet; t, triplet; q, quartet; m, multiplet; br, broad.

1. **Experimental details**

**Synthesis of complex 1.** A solution of [1,10-phenanthroline-5,6-diamino (0.12 mmol) and the dichloro-bridged [Ir(tpy)_2_Cl]_2_ (0.056 mmol) in dichloromethane (4 mL) and methanol (4 mL) was stirred at 65 °C overnight. After the reaction completed, an excess of solid ammonium hexafluorophosphate (NH_4_PF_6_) was added and stirred for another 0.5 h at room temperature. The solvent was removed under reduced pressure and the residue was purified by silica gel column chromatography (eluent, methanol/ dichloromethane, 1/20, v/v) to yield **1** as an orange powder. Yield: 50%. ^1^H NMR (400 MHz, DMSO-*d*_6_) δ 8.89 (dd, *J* = 8.0, 1.3 Hz, 2H), 8.22 (d, *J* = 8.1 Hz, 2H), 8.07 (dd, *J* = 5.4, 1.3 Hz, 2H), 7.95 – 7.89 (m, 4H), 7.84 (d, *J* = 8.0 Hz, 2H), 7.65 (d, *J* = 5.7 Hz, 2H), 7.09 (d, *J* = 6.1 Hz, 2H), 6.87 (d, *J* = 7.9 Hz, 2H), 5.99 (s, 2H), 2.08 (s, 6H), 1.62 (s, 4H). ^13^C NMR (100 MHz, CD_3_CN-*d*_3_) δ 148.70, 146.56, 137.76, 132.06, 130.55, 124.95, 124.48, 124.42, 123.02, 122.37, 118.92, 20.43. MALDI-TOF-HRMS: Calcd. for C_36_H_30_IrN_6_[M–PF_6_]^+^: 739.2161, found: 739.2128. Anal. (C_36_H_30_N_6_IrPF_6_) C, H, N: calcd 48.92, 3.42, 9.51; found 48.69, 3.40, 9.58.

**Figure S1.** Synthesis of complex **1**.

**Photophysical measurement.** Emission spectra and lifetime measurements for complexes were performed on a PTI TimeMaster C720 Spectrometer and a Horiba fluorescence spectrometer (FL3C-21) respectively. Error limits were estimated: λ (±1 nm); τ (±10%); φ (±10%). All solvents used for the lifetime measurements were degassed using three cycles of freeze-vac-thaw.

1. **Supporting information**

**Table S1.** Comparison of different fluorescent probes for NO detection.

| **Ref.** | **Probe** | **Response time** | **Linear response** | **Detection limit** | **Application** |
| --- | --- | --- | --- | --- | --- |
| 13 | Organic dye | 10 min | 0.4–3.4 μM | 0.084 μM | Raw 264.7 cells and Hippocampal tissue |
| 14 | Organic dye | 15 min | NA | 3 nM | Bovine aortic endothelial cells |
| 15 | Organic dye | 30 min | NA | NA | NA |
| 16 | Organic dye | 3 min | NA | NA | Rat kidney |
| 24 | Cu-based probe | 5 min | NA | NA | Raw 264.7 cells |
| 25 | Cu-based probe | 5 min | NA | NA | NA |
| 27 | Ir-based probe | 1 min | NA | NA | HeLa cells |
| This work | Ir-based probe | 1 min | 5–25 μM | 0.18 μM | HeLa cells |

**Table S2.** Photophysical properties of iridium(III) complex **1**.

| Complex | Quantum yield | λ_ex_ / nm | λ_em_ / nm | Lifetime / ns | UV-Vis absorption  λ_abs_ / nm (ε / dm^3^ mol^–1^ cm^–1^) |
| --- | --- | --- | --- | --- | --- |
| **1** | 0.19 | 355 | 608 | 265 | 240 (0.16× 10^5^), 300 (0.99× 10^4^), 368 (0.28× 10^4^) |


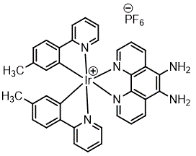

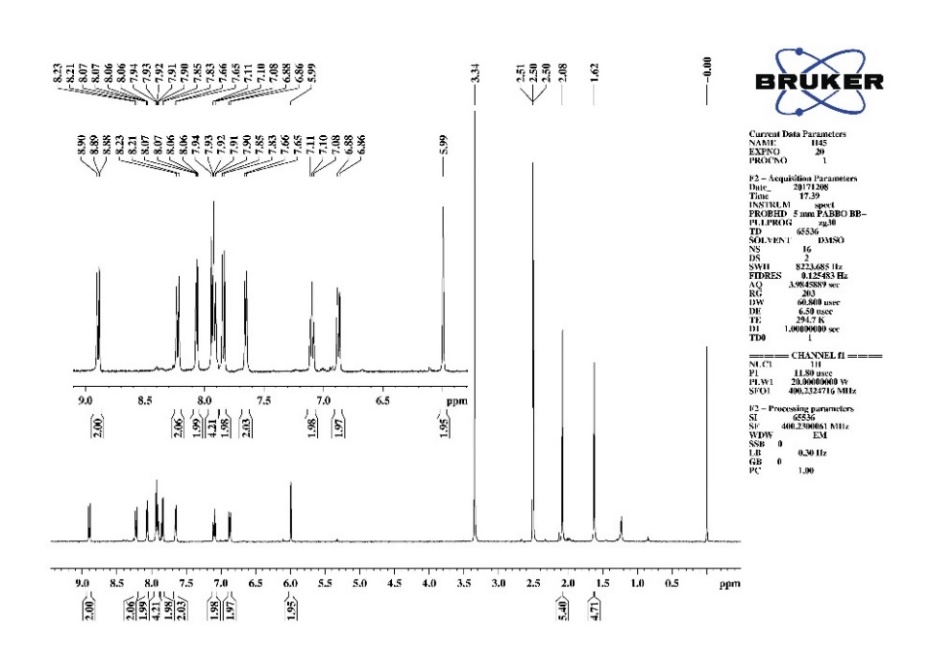


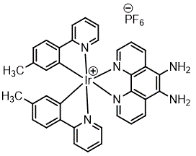

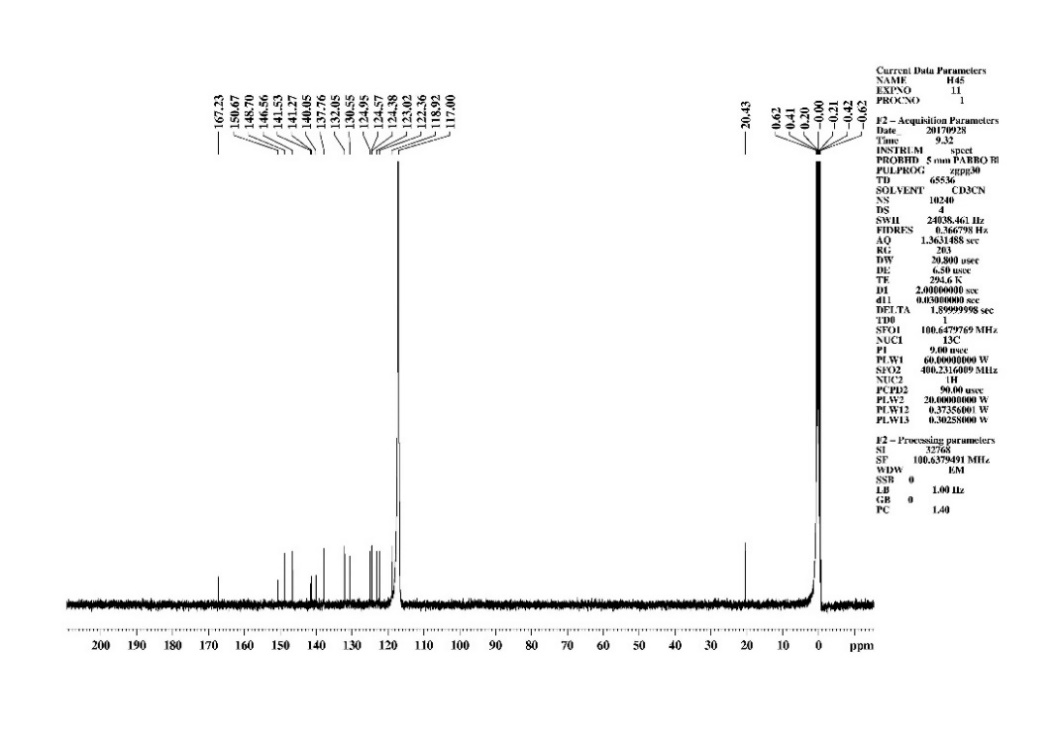


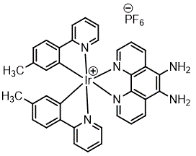

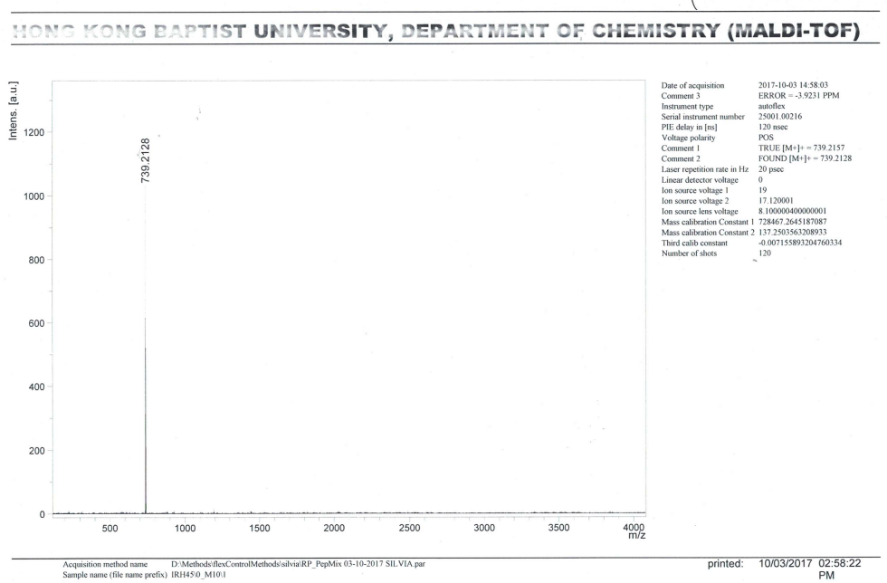


**Figure S2**. ^1^H NMR,^13^C NMR and HRMS spectra of complex **1**.


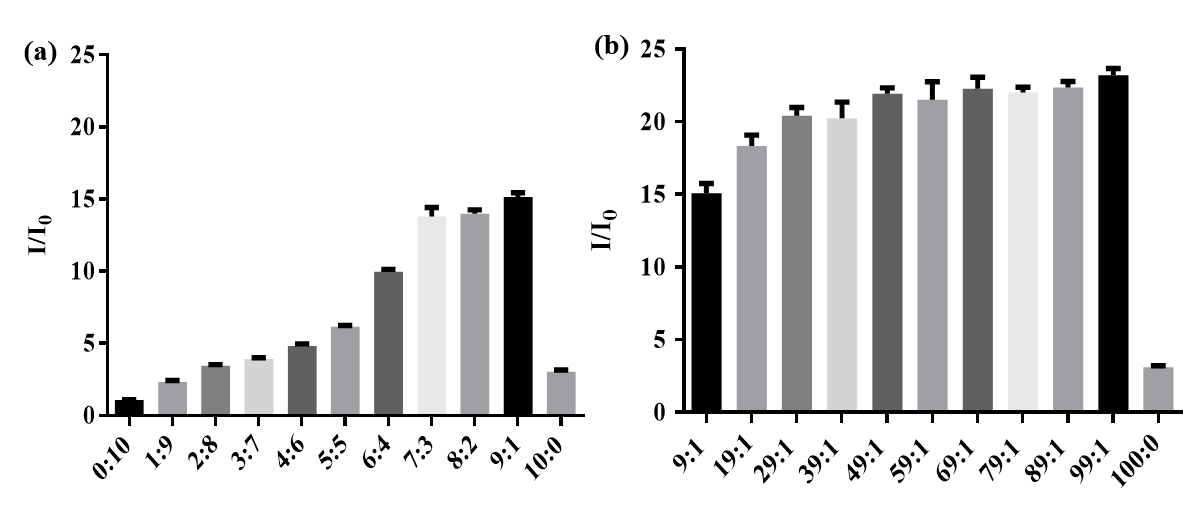


**Figure S3.** **(a)** Luminescence enhancement of complex **1** (5 μM) with SNP (25 μM) in various ratios of DMSO/PBS buffer (50 mM, pH = 7.4). **(b)** Luminescence enhancement of complex **1** (5 μM) with SNP (25 μM) in various ratios of DMSO/PBS buffer from 9:1 to 100:0 (50 mM, pH = 7.4).


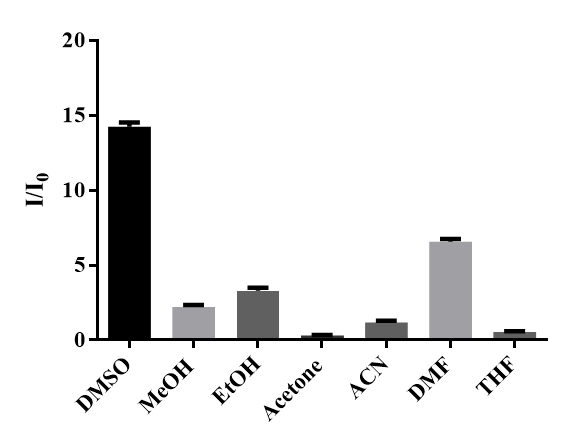


**Figure S4.** Luminescence enhancement of complex **1** (5 μM) with SNP (25 μM) in various types of organic solvents with 10% PBS buffer (50 mM, pH = 7.4).


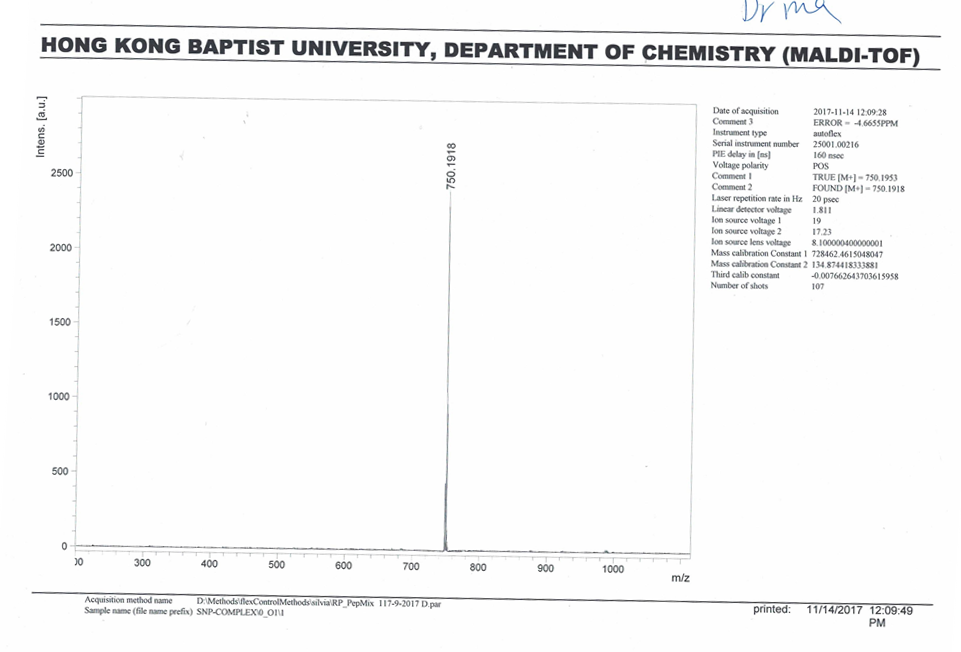


**Figure S5.** HRMS spectrum of the desired product complex **2**.


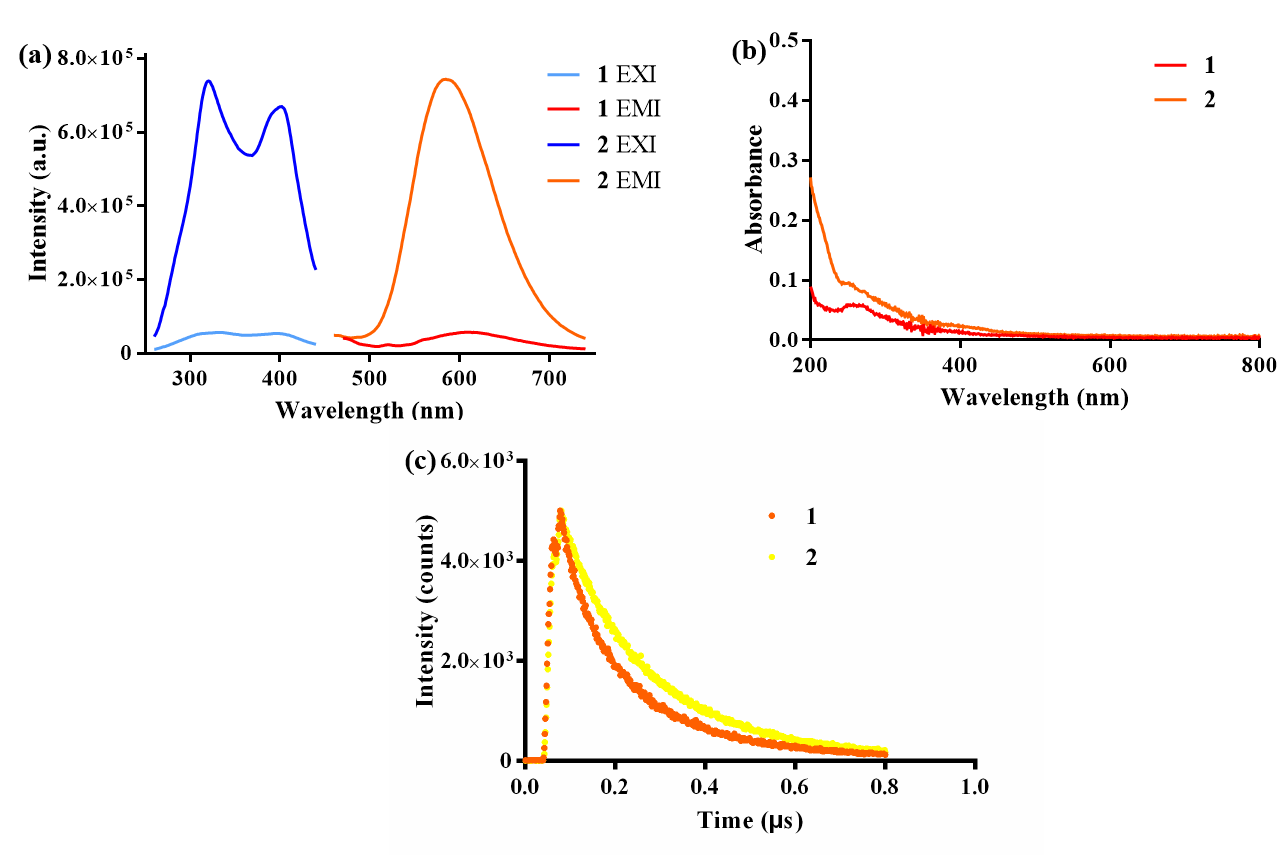


**Figure S6. (a)** Excitation and emission spectra of complexes **1** and **2** (5 μM) in DMSO. **(b)** UV/Vis absorption spectra of complexes **1** and **2** (5 μM) in acetonitrile. **(c)** Lifetime of complexes **1** and **2** (10 μM) in DMSO/PBS (9:1, v/v). λ_ex_ = 355 nm. τ**_1_** = 265 ns and τ**_2_** = 341 ns.


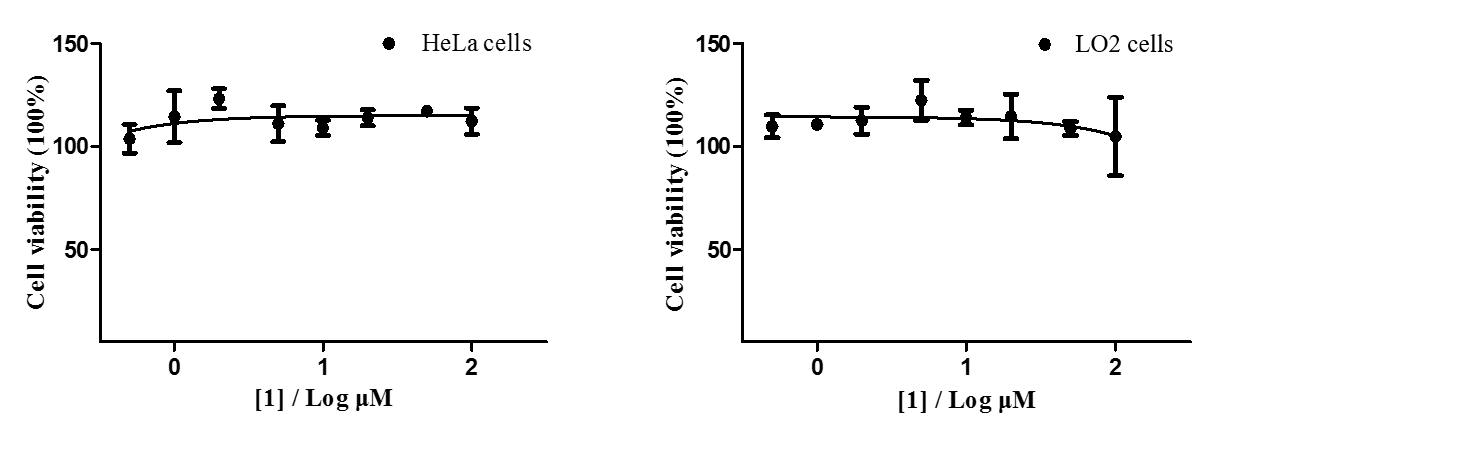


**Figure S7.** HeLa cells and LO2 cells were treated with complex **1** at the concentration of 0‒100 μM for 6 h at 37 °C. Complex **1** inhibited cell viability of HeLa cells and LO2 cells with IC_50_ values > 100 μM.


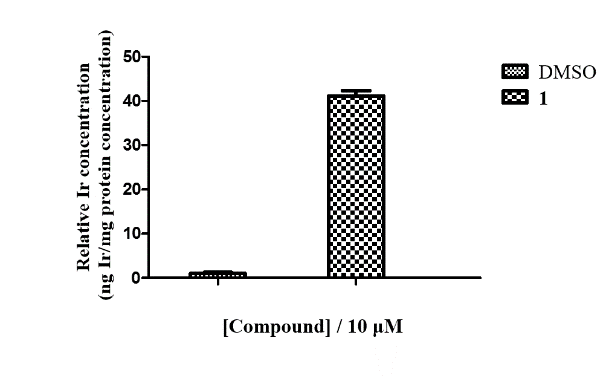


**Figure S8.** ICP-MS assay for iridium accumulation in HeLa cells. HeLa cells were incubated with complex **1** (10 μM) for 3 h at 37 °C. The iridium counts were normalized to protein concentration of organelles, which was determined by a BCA assay.


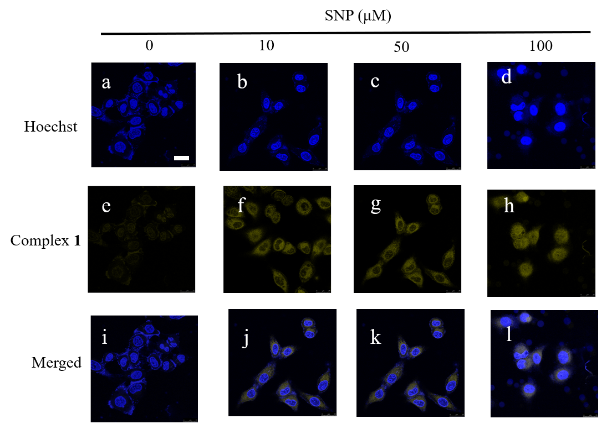


**Figure S9.** Confocal imaging of HeLa cells incubated with complex **1** (10 μM) with SNP (0‒100 μM) for 3 h at 37 °C after UV irradiation for 10 min. Excitation was at 405 nm, and luminescence images were recorded from 570 to 640 nm. Nuclei were stained with 5 μg/mL Hoechst for 5 min. The scale bar is 25 μm.


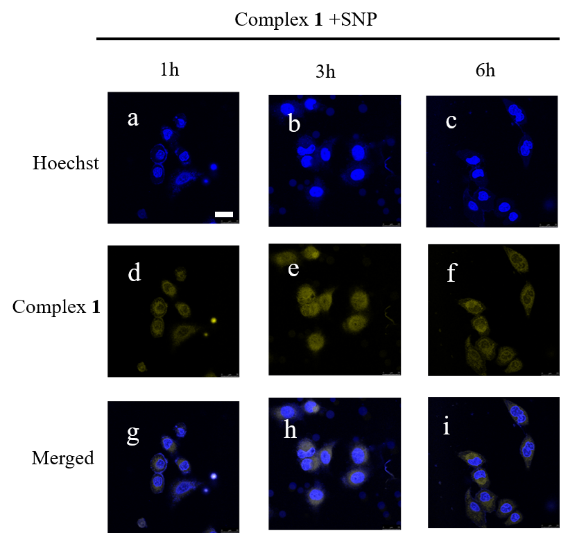


**Figure S10.** Confocal imaging of HeLa cells incubated with complex **1** (10 μM) with SNP (100 μM) for 1, 3, 6 h at 37 °C after UV irradiation for 10 min. Excitation was at 405 nm, and luminescence images were recorded from 570 to 640 nm. Nuclei were stained with 5 μg/mL Hoechst for 5 min. The scale bar is 25 μm.

1. **References**

1. K. Vellaisamy, G. Li, C.-N. Ko, H.-J. Zhong, S. Fatima, H.-Y. Kwan, C.-Y. Wong, W.-J. Kwong, W. Tan, C.-H. Leung, D.-L. Ma. *Chem. Sci.* 2018, **9**, 1119.

2. L.-J. Liu, W. Wang, S.-Y. Huang, Y. Hong, G. Li, S. Lin, J. Tian, Z. Cai, H.-M. D. Wang, D.-L. Ma, C.-H. Leung, *Chem. Sci.*, 2017, **8**, 4756.

3. J.-B. Liu, C. Yang, C.-N. Ko, K. Vellaisamy, B. Yang, M.-Y. Lee, C.-H. Leung and D.-L. Ma, *Sens. Actuators. B Chem.*, 2017, **243**, 971.

4. X. Chen, X. Tian, I. Shin and J. Yoon, *Chem. Soc. Rev.*, 2011, **40**, 4783.
